# Association of Testosterone-Related Dietary Pattern with Testicular Function among Adult Men: A Cross-Sectional Health Screening Study in Taiwan

**DOI:** 10.3390/nu13010259

**Published:** 2021-01-18

**Authors:** Adi-Lukas Kurniawan, Chien-Yeh Hsu, Jane C-J Chao, Rathi Paramastri, Hsiu-An Lee, Pao-Chin Lai, Nan-Chen Hsieh, Shu-Fang Vivienne Wu

**Affiliations:** 1 Research Center for Healthcare Industry Innovation, National Taipei University of Nursing and Health Sciences, 365 Ming-Te Road, Beitou District, Taipei 112, Taiwan; nchsieh@ntunhs.edu.tw (N.-C.H.); shufang@ntunhs.edu.tw (S.-F.V.W.); 2 Department of Information Management, National Taipei University of Nursing and Health Sciences, 365 Ming-Te Road, Beitou District, Taipei 112, Taiwan; cyhsu@ntunhs.edu.tw; 3 School of Nutrition and Health Sciences, College of Nutrition, Taipei Medical University, 250 Wu-Hsing Street, Xinyi District, Taipei 110, Taiwan; rara.paramastri@gmail.com; 4 Master Program in Global Health and Development, College of Public Health, Taipei Medical University, 250 Wu-Hsing Street, Xinyi District, Taipei 110, Taiwan; 5 Nutrition Research Center, Taipei Medical University Hospital, 252 Wu-Hsing Street, Xinyi District, Taipei 110, Taiwan; 6 Department of Computer Science and Information Engineering, Tamkang University, 151 Yingzhuan Road, Tamsui District, New Taipei 251, Taiwan; billy72325@gmail.com; 7 Department of Nursing, Shin Kong Wu Ho-Su Memorial Hospital, 95 Wen Chang Road, Shilin District, Taipei 111, Taiwan; emily500903@yahoo.com.tw; 8 Department of Nursing, Lotung Poh-Ai Hospital, 83 Nanchang Street, Luodong Township, Yilan County 265, Taiwan; 9 College of Nursing, School of Nursing, National Taipei University of Nursing and Health Sciences, 365 Ming-Te Road, Beitou District, Taipei 112, Taiwan

**Keywords:** dietary patterns, testicular function, sperm quality, male sex hormones, reduced-rank regression

## Abstract

Diets could play an important role in testicular function, but studies on how adherence to the dietary patterns influences human testicular function in Asian countries are scarce. Herein, we examined the association between testosterone-related dietary patterns and testicular function among adult men in Taiwan. This cross-sectional study recruited 3283 men who attended a private medical screening program from 2009 to 2015. Testosterone-related dietary pattern was generated by the reduced rank regression (RRR) method. The association between adherence to quartile of dietary pattern scores with sex hormones (testosterone, follicle-stimulating hormone (FSH), luteinizing hormone (LH), and estradiol (E2)) and sperm quality (sperm concentration (SC), total sperm motility (TSM), progressive motility (PRM), and normal sperm morphology (NSM)) were examined by multivariable linear regression. Hemoglobin (β = 0.57, *p* < 0.001), hematocrit (β = 0.17, *p* = 0.002), triglyceride (β = −0.84, *p* < 0.001), HDL-cholesterol (β = 3.58, *p* < 0.001), total cholesterol to HDL-cholesterol ratio (β = −0.78, *p* < 0.001), and uric acid (β = −10.77, *p* < 0.001) were highly correlated with testosterone levels. Therefore, these biomarkers were used to construct a testosterone-related dietary pattern. Highest adherence (Q4) to dietary pattern scores were negatively associated with lower testosterone in the pooled analysis (β = −0.89, *p* = 0.037) and normal-weight men (β = −1.48, *p* = 0.019). Likewise, men in the Q4 of the dietary pattern had lower SC (β = −5.55, *p* = 0.001) and NSM (β = −2.22, *p* = 0.007) regardless of their nutritional status. Our study suggesting that testosterone-related dietary pattern (rich in preserved vegetables or processed meat or fish, deep-fried foods, innards organs, rice or flour products cooked in oil, and dipping sauce, but low in milk, dairy products, legumes, or beans, and dark or leafy vegetables) was associated with a poor testicular function.

## 1. Introduction

Testosterone is the most potent male sex hormone, which plays important roles not only in reproductive function but also in male body composition and appearance. Testosterone is mostly produced by the Leydig cells (95%) of testes and it is controlled by the hypothalamic–pituitary–gonadal axis through luteinizing hormone (LH) as the key hormonal signals [1]. Whereas, sperm maturation and production are under the control of follicle-stimulating hormone (FSH) [2]. Under some circumstances, the release of testosterone can be converted to estradiol via adipose tissue-derived aromatase, which in turn acts as negative feedback inhibition of gonadotropin-releasing hormone (GnRH) and LH secretion [3,4]. It has been known also that testosterone has an effect to stimulate spermatogenesis and support the development of immature spermatozoa [1].

During the past few decades, there has been reported that semen quality showing a significant decline across the world. According to the current meta-regression study reported that sperm concentration (SC) and total sperm count (TSC) declined by 50–60% in total between 1973 and 2011 particularly among Western countries [5]. In spite of socio- and lifestyle factors such as age, obesity, smoking, alcohol consumption, and physical activity that can affect the testosterone levels and sperm quality, [6,7,8,9,10] accumulating evidence indicates that diet could play a crucial role in influencing male sex hormone and sperm quality [11,12]. Recent studies conducted in Western countries similarly reported that adherence to a healthy prudent diet has a better testicular function (higher total sperm count or concentration and testosterone levels) [11,12]. While the unhealthy western type of diet has detrimental effects on semen quality as well as sex hormones [13].

Dietary pattern is multiple approaches to investigate the synergistic effects and collinearity of food or nutrition with the disease risk. Using dietary patterns is a useful approach for describing the overall eating pattern of the population because people do not eat single isolated nutrients or food but they eat meals that consist of a variety of food with combinations of complex nutrients [14]. Reduced rank regression (RRR) is a combination of hypothesis-driven method (priori) and data-driven method (posteriori) for deriving dietary patterns [15]. RRR uses a linear combination of predictor variables (food items) and response variables (biomarkers) based on prior knowledge to generate dietary patterns related to disease [15]. However, based on our knowledge, no study has been performed by using both posteriori and priori approaches to examine the effects of testosterone-related dietary patterns on sperm quality and limited studies have been conducted in Asian countries. Likewise, previous studies investigating the effects of diet on the sex hormone were conducted in a relatively modest sample size and the results were not entirely consistent. Based on these premises, we aimed to investigate the association of testosterone-related dietary patterns generated by RRR on testicular function among adult men in Taiwan. In this study, we tried to stratify the subjects between normal and overweight or obese men, because overweight and obesity have been known to pose decisive effects on testicular function.

## 2. Materials and Methods

### 2.1. Study Participants Recruitments

This cross-sectional study used the database collected by the private institution of Mei Jau Health Management (MJHM) from 2009 to 2015. The MJHM has four health check-up locations across Taiwan (Taipei, Taoyuan, Taichung, and Kaohsiung), which provides annual health examinations to its members. Prior to the physical and blood or urine tests, participants completed a validated self-reported written questionnaire to report their sociodemographic, lifestyle, medical history, and dietary habit data. All participants had signed a consent form to process the data for research purposes only and without personal identification. Initially, we retrieved 9276 healthy men without any type of cancers, thyroid disorders, hepatitis, and liver cirrhosis. After excluded men with hypertension (*n* = 237), diabetes (*n* = 56), multiple entries (*n* = 2574), and men without testosterone or sperm parameters data (*n* = 3126), in total 3283 men were included in this study. The Taipei Medical University Joint Institutional Review Board (TMU-JIRB N202010035) approved this study.

### 2.2. Clinical and Biochemical Data Measurements

An auto-anthropometers (Nakamura KN-5000A, Tokyo, Japan) measures the body weight and height of men during the health examination. Body mass index (BMI) was calculated according to weight (kg) divided by the square of height (m). Nutrition status based on BMI was defined as follows: normal (18.5 kg/m^2^ ≤ BMI < 24 kg/m^2^), overweight (24 kg/m^2^ ≤ BMI < 27 kg/m^2^), and obese (BMI ≥ 27 kg/m^2^) [16]. Prior to the blood measurements, all participants were asked to fast for at least 8 h. Blood counts such as leukocytes, neutrophils, lymphocytes, erythrocytes, hemoglobin, and hematocrit (Abbott Cell-Dyn 3700, Abbott Park, IL, USA) were measured in each man. Moreover, fasting glucose, triglycerides, total cholesterol, HDL-cholesterol, LDL-cholesterol C-reactive protein, iron, total iron-binding capacity, transferrin saturation, ferritin, and uric acid (Toshiba C8000 auto-analyzer, Tokyo, Japan) were analyzed at the MJHM central laboratory. Whereas serum creatinine levels were analyzed by uncompensated Jaffe method with alkaline picrate kinetic test.

Men’s sex hormones such as follicle-stimulating hormone (FSH), luteinizing hormone (LH), total testosterone, and estradiol (E2) were measured by chemiluminescent immunoassay (CMIA Architect Abbott, IL, USA). Sperm parameters were collected via masturbation after at least 3 days of abstinence and the sample was analyzed within one hour. Four sperm parameters were used in this study including sperm concentration (SC), total sperm motility (TSM), progressive motility (PRM), and normal sperm morphology (NSM). SC was measured using a hemocytometer (Hauser Scientific Inc., Horsham, PA, USA). Sperm motility was classified as PRM for WHO class A + B and TSM for WHO class A + B + C [17]. The quality control and calibration techniques were performed by the laboratory and supplied by the manufacturer, with the coefficient of variation ranged less than 3%.

### 2.3. Dietary Pattern Assessments and Other Covariates

Food consumption habits were collected by using a standardized and validated semi-quantitative food frequency questionnaire (FFQ) [18]. The FFQ consists of twenty-two food groups that participants need to answer on how often they consumed certain portions of each food group in the past month. The frequency of intake has five response options ranged from daily or weekly together with the definition of the portion size (bowl, glass, or serving). For example, the questions for the consumption related to vegetables (light or dark leafy colored vegetables, vegetables with oil or salad dressing, and root crops) had 5 response options: <0.5 bowls/day, 0.5–1 bowls/day, 1–1.5 bowls/day, 1.5–2 bowls/day, and 2 bowls/day (1 bowl = 11 cm in diameter). For milk or beverages, the 5 response options were: none or less than 1 glass/week, 1–3 glasses/week, 4–6 glasses/week, 1 glass/day, and 2 or more glasses/day (1 glass = 240 mL). For the intake of other food items, the 5 response options were: <1 serving/week, 1–3 servings/week, 4–6 servings/week, 1 serving/day, and ≥2 servings/day. Detailed information about dietary assessment has been published elsewhere [18].

Other covariates including age and lifestyle behaviors (smoking, alcohol drinking, and physical activity) were also collected from the database. We classified the age into 3 groups: <30 years, 31–40 years, and >41 years. Moreover, smoking and alcohol drinking were dichotomized as “no” if participants reported never smoke or drink and “yes” if participants reported as current/past smokers or drinking ≥ 1 to 2 times/week. Physical activity was assessed by self-reporting intensity (no, light, moderate, heavy, and intense) of doing exercise in the last two weeks.

### 2.4. Data Analysis

Continuous and categorical variables are presented as a mean ± standard deviation (SD) and number (percentage) respectively. A general linear model was used for comparing the mean difference in the continuous variables while the chi-square test was performed for comparing the categorical variables according to nutritional status. A multivariable linear regression presented as beta (β) coefficient regression and 95% confidence intervals (CIs), was performed to identify the biomarkers that related to testosterone and to examine the association between quartiles of dietary pattern scores with sex hormone parameters (FSH, LH, testosterone, E2) and sperm parameters (SC, TSM, PRM, and NSM). Two models of adjustment were used in this study; model 1 was adjusted by age and BMI while model 2 was adjusted for all the confounders (age, BMI, smoking status, alcohol drinking status, physical activity, and fasting glucose). All analysis above was performed by using STATA version 13 (StataCorp LP, College Station, TX, USA).

**Figure 1:**
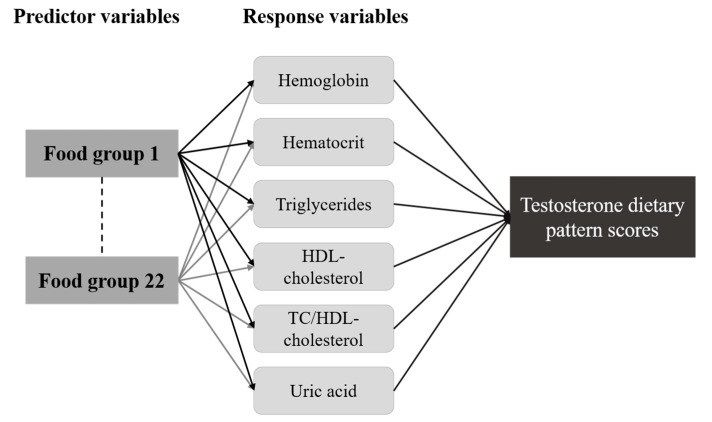
Schematic diagram of reduced rank regression conceptual framework. HDL-cholesterol, high-density lipoprotein cholesterol; TC/HDL-cholesterol, total cholesterol to HDL-cholesterol ratio.

Testosterone related dietary pattern was identified using the reduced rank regression (RRR) method in SAS 9.4 (SAS Institute Inc., Cary, NC, USA) with “proc pls” function as described previously [19]. By using RRR, we able to identify a linear combination of the predictor variables, which are associated with selected response variables and likely to include dietary patterns related to the disease of interest [15]. The predictor variables refer to the food groups, which are 22 food groups derived from the FFQ while the response variables refer to the nutrients or blood biomarkers. In our study, we identified 6 biomarkers that significantly correlated to testosterone levels (Figure 1). Moreover, the number of dietary factors that RRR generates depends on how many response variables are used. However, out of six factors, we only retained the first dietary factor for the analysis as it explained the largest amount of variation in the response variables. The absolute value of factor loading ≥ 0.20 was selected to identify a testosterone-related dietary pattern. A *p*-value of <0.05 was considered statistically significant.

## 3. Results

### 3.1. Characteristics of Study Participants According to Nutritional Status

Table 1 presents the characteristics of men according to nutritional status. More than half of men had none or light physical activity. Overweight or obese men had higher blood count parameters, fasting glucose, and blood lipids compared to normal-weight men. Moreover, men who were overweight or obese had lower kidney function (eGFR: 89.2 ± 13.1 mL/min/1.73 m^2^), testosterone levels (15.8 ± 5.4 nmol/L), and SC (45.0 ± 24.9×10^6^/mL) compared to normal-weight men.

### 3.2. Association of Serum Testosterone with Selected Biochemical Data

A fully adjusted multivariable linier regression showed that every 1 g/dL increment in hemoglobin, 1% increase in hematocrit, and 1 mmol/L increase in HDL-cholesterol were associated with elevated of 0.57 (95% CI: 0.25, 0.89, *p* < 0.001), 0.17 (95% CI: 0.06, 0.28, *p* = 0.002), and 3.58 (95% CI: 2.45, 4.71, *p* < 0.001) nmol/L of testosterone levels, respectively. In contrast, triglycerides (β = −0.84, *p* < 0.001), TC/HDL-cholesterol ratio (β = −0.78, *p* < 0.001), and uric acid (β = −10.77, *p* < 0.001) were inversely associated with testosterone levels (Table 2). Moreover, only sex hormone of FSH (β = −0.12, *p* = 0.002) and E2 (β = 0.05, *p* < 0.001) had significant association with testosterone levels.

### 3.3. Testosterone-Related Dietary Pattern

Testosterone-related dietary pattern was identified by using the RRR method. Response variables were selected based on the association between testosterone and the independent variables, which were hemoglobin, hematocrit, HDL-cholesterol, triglycerides, TC/HDL-cholesterol ratio, and uric acid (Table 2). Table 3 shows that the food group of milk, dairy products, legumes or beans products, and dark or leafy vegetables were negatively correlated with the dietary pattern scores (factor loading ≤ −0.20). Meanwhile, innards organs, rice or flour products cooked in oil, deep-fried foods, dipping sauce, and preserved vegetables or processed meat or fish were positively correlated with dietary pattern scores (factor loading ≥ 0.20). The cumulative variation explained by testosterone-related dietary pattern was 29.65%. The six response variables were explained as 2.72% for the total variation and largely driven by hemoglobin (1.91%), hematocrit (1.86%), and TC/HDL-cholesterol ratio (1.76%). Characteristics of men according to quartiles of dietary pattern scores are shown in Table S1.

### 3.4. Association of Dietary Pattern with Sex Hormone and Sperm Parameters in Men

Dietary pattern scores were stratified into quartiles and Table 4 demonstrated the association of each quartile of dietary pattern scores with sex hormone biomarkers according to nutritional status. In the pooled analysis, the fully adjusted model showed that the highest adherence to the dietary pattern scores (Q4) was associated with lower testosterone levels (β = −0.89, 95% CI: −1.73, −0.05; *p* = 0.037) compared to the lowest dietary pattern scores (Q1: Ref). Similarly, when we stratified the men based on their nutritional status, normal-weight men with the highest dietary pattern scores (Q4) had lower testosterone levels (β = −1.48, 95% CI: −2.72, −0.24; *p* = 0.019) compared to the lowest dietary pattern scores (Q1). There was no significant association between quartiles’ dietary pattern scores with other sex hormones both in pooled and stratified analysis.

Table 5 shows the association between quartile scores of dietary pattern with sperm parameters. Pooled analysis showed that highest adherence of the dietary pattern scores (Q4) was associated with lower SC (β = −5.55, 95% CI: −8.68, −2.41; *p* = 0.001) and NSM (β = −2.22, 95% CI: −3.83, −0.62; *p* = 0.007) compared to men with lowest quartile (Q1). Similarly, based on nutritional status, normal and overweight or obese men in the highest quartile (Q4) had SC reduced by 4.61 × 10^6^/mL and 6.73 × 10^6^/mL, respectively, compared with men in the lowest quartile (Q1). Their NSM was also reduced by 2.23% and 2.90% both in the normal and overweight/obese men in the highest quartile of dietary pattern scores (Q4).

## 4. Discussion

Based on our knowledge, the present study is the first study using a larger sample size to identify a testosterone-related dietary pattern, generated by the RRR method, in relation to testicular function. Overall, we identified the response variables for RRR based on the significant linear association between testosterone and other specific biomarkers. Our study found that hemoglobin, hematocrit, fasting blood glucose, triglycerides, HDL-cholesterol, TC/HDL-cholesterol ratio, uric acid, FSH, and E2 were significantly correlated with testosterone levels. It is recognizable that testosterone levels are affected by age, BMI, and other comorbidities such as the presence of type 2 diabetes [20]. Thus, we did not use fasting blood glucose as one of the response variables when generating the dietary pattern in the RRR method. Moreover, other sex hormones that related to testosterone such as FSH and E2 were also not included in the response variables due to their distinct relationship with testosterone levels.

Several previous studies have been documented the strong association between testosterone levels and hemoglobin and hematocrit [21,22,23]. Two randomized controlled trials among older men in agreement reported that testosterone administration significantly increased the hemoglobin and hematocrit levels in accordant with increased erythropoietin (EPO) levels [21,22]. There are several concepts on how testosterone treatment causes an increase in hemoglobin and hematocrit as well as red blood cell (RBC) volume. First, testosterone has the ability to increase and stimulate EPO release, a protein produced by the kidney, and directly stimulates the bone marrow for RBC synthesis [21]. Second, testosterone may suppress hepcidin, a hormone that regulates iron utilization, leading to an increase in iron absorption for erythropoiesis [21]. In the present study, we also found that triglycerides and TC/HDL-cholesterol ratio had a negative association with testosterone levels while HDL-cholesterol was positively associated with testosterone levels. Our findings are consistent with the results of previous studies that demonstrated that a higher TG/HDL-cholesterol index was significantly associated with low testosterone levels in middle-aged and elderly men [24,25]. Previous studies used TG/HDL-cholesterol index as a marker of insulin resistance (IR) and suggested the inverse relationship between testosterone and IR [24,25,26]. Low testosterone levels together with an increase of abdominal fat mass and a decrease of lean muscle mass trigger free fatty acid influx to the liver, pro-inflammatory production, and dysfunction of muscle mitochondria oxidative phosphorylation, which contributes to the degree of IR [24,27]. In the IR state, GnRH activation may impair, which causes a decline in the LH secretion [27]. Several epidemiological studies also have shown the inverse relationship between uric acid and testosterone levels [28,29]. There are plausible mechanisms for the significant association between uric acid and testosterone levels in men. First, IR subsequently caused by low testosterone levels, resulting in reduced secretion of uric acid in renal tubular epithelial cells and the renal excretion of uric acid [30]. Second, due to testosterone function to promote the synthesis of protein and nucleic acids, low testosterone levels may ameliorate the synthesis of endogenous purine, which causes hyperuricemia [30].

Our findings revealed that testosterone-related dietary pattern was characterized by high intakes of innards organs, rice or flour products cooked in oil, deep-fried foods, preserved vegetables, processed meat or fish, and dipping sauce but low intakes in milk, dairy products, legumes or beans products, and dark or leafy vegetables. Men in greatest adherence to the testosterone-related dietary pattern were associated with lower testosterone levels. In accordance with the role of testosterone in affecting sperm maturation and development, our study also found that testosterone-related dietary pattern decreased the SC and percentage of NSM, suggesting a reduction in spermatogenesis. However, a recent study conducted among 2935 Danish men showed reversed results [11]. The authors reported that men with the highest adherence to the Western pattern had higher testosterone levels compared to men with less adherence. Nevertheless, the FSH and LH levels have remained unchanged regardless of the adherence to the dietary patterns [11]. In our study, it appears that higher quartiles of the testosterone-related dietary pattern may lead to increased aromatization of testosterone to estradiol (Table S1). We hypothesize that this condition may result in negative feedback at the hypothalamic–pituitary–gonadal axis level. Thus, it might also explain why FSH and LH were unchanged as a compensation system. Therefore, we can speculate that adherence to the testosterone-related dietary pattern at least partly leads to a reduction in spermatogenesis.

In the present study, we used a hybrid method (priori and posteriori approached) of RRR to generate a dietary pattern based on a significant association of selected biomarkers (response variables) with testosterone levels. Our findings were similar to our previous study that using *priori* approached based on previous knowledge to generate a pattern [31]. Our previous study found that a “Western diet” and “high carbohydrate diet” were significantly associated with linear declines of SC and NSM. However, several limitations need to be addressed concerning using priori approaches. First, this method limits the ability to distinguish the correlation between food items and the pattern as well as with the biomarkers. Second, there is a subjective selection of the pattern components and cut-offs [32]. For example, in the previous study, we included milk and dairy products as a definition of the Western diet. In contrast, the present study showed that milk and dairy products negatively correlated with the dietary pattern meaning that they donated a reduction in the dietary score. Nevertheless, studies on the relationship between milk and dairy products intake and testicular function are inconclusive. Intake of dairy products, particularly full-fat dairy products has been related to lower testicular function [33]. Meanwhile, a longitudinal study showed the converse: the intake of low-fat dairy products was associated with higher SC and better sperm motility [34]. A plausible explanation could be the presence of type 1 insulin growth factor (IGF-1) in Leydig cells for regulating Sertoli cell proliferation, meaning that spermatogenesis is a process that requires insulin and IGF-1, and low-fat dairy intake may be associated with elevated levels of IGF-1 [13,35]. Although the evidence largely showed the benefit of low fat versus full-fat dairy products, more randomized trial studies are needed to support the findings.

Our study also reported that high intakes of a “high-cholesterol fat diet: innards organs, deep-fried food, processed meats” were associated with lower testicular function, particularly low testosterone levels, SC, and NSM. Similarly, previous studies also reported that higher amounts of processed meat intake were inversely related to sperm quality [36,37]. High saturated fatty acids, especially trans fatty acids and hormone residues that present in the Western type of diet are the major causes of such negative effects [35]. Increased intake of a high-fat diet leads to leptin resistance that has been associated with impaired spermatogenesis and dysfunction of the hypothalamic-pituitary axis [35]. Furthermore, elevated plasma cholesterol by a fat-rich diet is believed to promote endocytic accumulation of tight junction in Sertoli cells and mitigate the integrity of the blood-testes barrier, thus contributing to impaired spermatogenesis [35,38]. Recently, previous studies also have shown that the small non-coding RNA (sncRNA) in the sperm are susceptible to paternal nutrition, and exposure to different diets such as high-fat diets and high sugar diets may influence the offspring’s sperm function [39,40]. In contrast, a diet rich in vegetables, nuts, and beans is thought to improve testicular function [41,42]. Vegetables, legumes, or beans are rich in antioxidants including poly-unsaturated fatty acid (PUFA), vitamin A, vitamin C, vitamin C, folic acid beta-carotene, minerals, and fibers that have a protection ability against reactive oxygen species and other free radicals; thus, reducing sperm DNA damage [13]. However, amounts of phytoestrogen in beans, particularly soybeans must be taken into consideration. The literatures on soy or soy-derived products and testicular function are still inconsistent and scarce. For example, a cross-sectional study among subfertile males found that higher consumption of soy-based foods inversely associated with sperm concentration [43]. In contrast, a randomized double-blind crossover design study did not demonstrate that soybean intake has been associated with the deterioration of testicular function [44]. A plausible argument is that Asian diets include phytoestrogens-rich foods without any possible detrimental effects on testicular function. However, more studies are needed to determine and validate the effect of the phytoestrogens-rich diet on fertility.

Based on our knowledge, our study is the first study using the RRR method to generate a testosterone-related dietary pattern in a large dataset. Previously, a study by Hu et al., [45] generated testosterone-associated dietary pattern from a relatively modest sample size (125 adult men). The authors used testosterone levels and insulin as response biomarkers and their adjustment models (age and BMI) were also modest. However, as testosterone levels are affected by diabetes and insulin levels, adjusting the findings with diabetes biomarkers are therefore necessary to avoid confounders and risk of bias. The strength of our study includes the larger sample size in the testicular function data that can represent the generalizability of the findings to the population. In addition, we used the RRR method to generate dietary patterns. The RRR method uses both food groups as predictor variables and biomarkers as response variables, which allow the researchers to generate a dietary pattern that specific to the disease of interest [15]. However, selecting response variables can be personally subjective. Our study has some limitations, including the potential misclassification bias of self-reported FFQ and the testicular function parameters that are only based on a single measurement. Semen samples were collected using self-home collection kits and were requested to be sent to the laboratory within one hour may not have equal quality as an on-site collection. Data availability of other sex hormone parameters including inhibin B and sex hormone-binding globulin (SHBG) are limited. Thus, the interpretation of the findings is less straightforward. Additionally, the cross-sectional study design limits our ability to determine causality. Finally, although we did adjust for some potential confounders, we cannot exclude other confounding factors such as energy or protein intake, occupational status, depression or stress, anxiety, prolonged exposure to high temperatures, environmental pollution, exposure to pesticides or chemical toxins (cadmium and lead), and radiation.

## 5. Conclusions

Our study suggesting that testosterone-related dietary pattern (rich in preserved vegetables or processed meat or fish, deep-fried foods, innards organs, rice or flour products cooked in oil, and dipping sauce, but low in milk, dairy products, legumes or beans, and dark or leafy vegetables) was associated with a poor testicular function (lower testosterone levels, SC, and NSM). Dietary modification may positively affect the quality of testicular function. Future longitudinal studies with prospective measurements such as randomized clinical trials are needed to confirm the study findings.

## Figures and Tables

**Table 1:** Characteristics of men according to nutritional status.

Variables	Total (*n* = 3283)	Nutritional Status		*p* ^a^
		Normal (*n* = 1756)	Overweight/Obese (*n* = 1527)	
Age				<0.001
<30 year	1130 (34.4)	643 (36.6)	487 (31.9)	
31–40 year	1527 (46.5)	828 (47.2)	699 (45.8)	
>41 year	626 (19.1)	285 (16.2)	341 (22.3)	
Smoker				0.001
No	2035 (62.0)	1135 (64.6)	900 (58.9)	
Yes	1248 (38.0)	621 (35.4)	627 (41.1)	
Alcoholic drinker				0.02
No	2584 (78.7)	1410 (80.3)	1174 (76.9)	
Yes	699 (21.3)	346 (19.7)	353 (23.1)	
Physical activity				0.28
None/light	1844 (56.2)	971 (55.3)	873 (57.2)	
Moderate/intense	1439 (43.8)	785 (44.7)	654 (42.8)	
BMI, kg/m^2^	24.1 ± 3.4	21.7 ± 1.7	26.8 ± 2.7	<0.001
Leukocytes, 10^3^/μL	6.2 ± 1.5	5.9 ± 1.5	6.5 ± 1.5	<0.001
Neutrophil/lymphocyte ratio	1.8 ± 0.8	1.7 ± 0.8	1.8 ± 0.8	0.003
Erythrocytes, 10^6^/μL	5.2 ± 0.4	5.1 ± 0.4	5.2 ± 0.4	<0.001
Hemoglobin, g/dL	15.4 ± 1.0	15.3 ± 0.9	15.4 ± 1.0	<0.001
Hematocrit, %	45.7 ± 2.8	45.5 ± 2.7	45.9 ± 2.8	<0.001
Fasting glucose, mmol/L	5.5 ± 0.7	5.4 ± 0.5	5.6 ± 0.8	<0.001
Triglycerides, mmol/L	1.4 ± 0.8	1.1 ± 0.6	1.6 ± 1.0	<0.001
Total cholesterol, mmol/L	5.0 ± 0.9	4.9 ± 0.8	5.2 ± 0.9	<0.001
HDL-cholesterol, mmol/L	1.3 ± 0.3	1.4 ± 0.3	1.3 ± 0.3	<0.001
LDL-cholesterol, mmol/L	3.1 ± 0.8	3.0 ± 0.8	3.2 ± 0.8	<0.001
TC/HDL-cholesterol ratio	3.9 ± 1.0	3.6 ± 0.9	4.2 ± 1.0	<0.001
Creatinine, μmol/L	95.3 ± 10.8	94.9 ± 10.7	95.8 ± 10.9	0.02
eGFR, mL/min/1.73 m^2^	90.2 ± 13.0	91.1 ± 12.9	89.2 ± 13.1	<0.001
C-reactive protein, nmol/L	19.7 ± 32.8	16.4 ± 29.0	23.5 ± 36.3	<0.001
Iron, μmol/L	18.7 ± 6.3	18.8 ± 6.4	18.5 ± 6.2	0.13
TIBC, μmol/L	55.7 ± 8.0	55.0 ± 7.7	56.5 ± 8.3	0.07
Transferrin saturation, %	45.0 ± 21.7	45.0 ± 21.8	45.0 ± 21.8	0.98
Ferritin, μg/L	240.7 ± 151.6	226.0 ± 128.5	252.9 ± 167.6	0.07
Uric acid, mmol/L	0.4 ± 0.1	0.4 ± 0.1	0.4 ± 0.1	<0.001
FSH, IU/L	4.6 ± 5.4	4.6 ± 7.0	4.6 ± 3.7	0.96
LH, IU/L	3.3 ± 2.2	3.4 ± 2.7	3.2 ± 1.7	0.54
Testosterone, nmol/L	17.5 ± 6.0	19.3 ± 6.1	15.8 ± 5.4	<0.001
E2, pmol/L	90.3 ± 32.8	88.4 ± 30.7	91.8 ± 34.3	0.16
SC, 10^6^/mL	46.6 ± 25.4	47.8 ± 25.8	45.0 ± 24.9	0.01
TSM, %	67.1 ± 11.6	67.2 ± 11.6	67.0 ± 11.6	0.81
PRM, %	48.3 ± 14.8	48.3 ± 14.7	48.3 ± 14.8	0.92
NSM, %	67.0 ± 13.3	67.4 ± 13.3	66.4 ± 13.3	0.09

BMI, body mass index; HDL-cholesterol, high-density lipoprotein cholesterol; LDL-cholesterol, low-density lipoprotein cholesterol; TC, total cholesterol; eGFR, estimated glomerular filtration rate; TIBC, total iron-binding capacity; FSH, follicle-stimulating hormone; LH, luteinizing hormone; E2, estradiol; SC, sperm concentration; TSM, total sperm motility; PRM, progressive motility; NSM, normal sperm morphology. Data are expressed in number (%) and mean ± standard deviation (SD) for categorical and continuous variables, respectively. ^a^ *p*-value was analyzed using a chi-square test for categorical variables and a general linear model for continuous variables.

**Table 2:** Multivariable linear regression of serum testosterone with biochemical variables.

Variables	Model 1 ^a^			Model 2 ^b^		
	β	95% CI	*P*	β	95% CI	*p*
Leukocytes, 10^3^/μL	0.04	−0.17, 0.25	0.69	−0.03	−0.24, 0.18	0.77
Neutrophil/lymphocyte ratio	−0.29	−0.66, 0.08	0.12	−0.28	−0.64, 0.07	0.12
Erythrocytes, 10^6^/μL	0.40	−0.35, 1.15	0.30	0.47	−0.26, 1.20	0.21
Hemoglobin, g/dL	0.61	0.29, 0.93	<0.001	0.57	0.25, 0.89	<0.001
Hematocrit, %	0.18	0.07, 0.29	0.002	0.17	0.06, 0.28	0.002
Fasting glucose, mmol/L	−0.94	−1.31, 0.58	<0.001	−0.93	−1.29, −0.56	<0.001
Triglycerides, mmol/L	−0.82	−1.16, −0.48	<0.001	−0.84	−1.18, −0.50	<0.001
Total cholesterol, mmol/L	0.18	−0.18, 0.54	0.33	0.18	−0.18, 0.53	0.33
HDL-cholesterol, mmol/L	3.86	2.73, 5.00	<0.001	3.58	2.45, 4.71	<0.001
LDL-cholesterol, mmol/L	0.12	−0.26, 0.50	0.52	0.14	−0.23, 0.51	0.47
TC/HDL-cholesterol ratio	−0.84	−1.21, −0.48	<0.001	−0.78	−1.15, −0.42	<0.001
Creatinine, μmol/L	0.03	−0.00, 0.06	0.07	0.02	−0.01, 0.05	0.19
eGFR, mL/min/1.73 m^2^	−0.03	−0.06, 0.00	0.07	−0.02	−0.05, 0.01	0.22
C-reactive protein, nmol/L	−0.01	−0.02, 0.00	0.17	−0.01	−0.01, 0.01	0.35
Iron, μmol/L	−0.02	−0.07, 0.03	0.47	−0.04	−0.09, 0.01	0.14
TIBC, μmol/L	−0.06	−0.16, 0.04	0.27	−0.06	−0.16, 0.04	0.24
Transferrin saturation, %	0.01	−0.03, 0.04	0.78	0.00	−0.04, 0.04	0.98
Ferritin, μg/L	0.00	−0.00, 0.00	0.77	0.00	−0.00, 0.00	0.66
Uric acid, mmol/L	−9.15	−13.81, −4.49	<0.001	−10.77	−15.31, −6.22	<0.001
FSH, IU/L	−0.11	−0.19, −0.04	0.004	−0.12	−0.19, −0.04	0.002
LH, IU/L	−0.05	−0.23, 0.13	0.60	−0.07	−0.25, 0.11	0.44
E2, pmol/L	0.05	0.04, 0.06	<0.001	0.05	0.04, 0.06	<0.001

HDL-cholesterol, high-density lipoprotein cholesterol; LDL-cholesterol, low-density lipoprotein cholesterol; TC, total cholesterol; eGFR, estimated glomerular filtration rate; TIBC, total iron-binding capacity; FSH, follicle-stimulating hormone; LH, luteinizing hormone; E2, estradiol. ^a^ Model 1:adjusted by age and BMI. ^b^ Model 2: adjusted by model 1 and smoker, alcoholic drinker, physical activity, and fasting blood glucose.

**Table 3:** Food groups that were associated with testosterone-related dietary pattern scores.

	Explained Variation (%)	Factor Loading ^a^
**Predictor food groups**		
Milk	26.43	−0.47
Dairy products	5.48	−0.21
Innards organs	16.12	0.37
Legumes or beans products	6.19	−0.23
Dark or leafy vegetables	6.44	−0.23
Rice or flour products cooked in oil	11.56	0.31
Deep-fried foods	8.78	0.27
Preserved vegetables, processed meat, or fish	8.44	0.27
Dipping sauce	9.24	0.28
Total explained variation (%)	29.65	
**Respond biomarkers**		
Hemoglobin	1.91	
Hematocrit	1.86	
Triglycerides	1.11	
HDL-cholesterol	0.68	
TC/HDL-cholesterol ratio	1.76	
Uric acid	1.26	
Total explained variation in biomarkers (%)	2.72	

^a^ Factor loadings are correlations between food groups and the dietary pattern scores (correlation coefficient cut off ≥|0.20|).

**Table 4:** Multivariate linear regression coefficients for sex hormones in relation to the dietary pattern quartiles according to nutrition status.

Dietary Pattern Quartiles	Testosterone, nmol/L		FSH, IU/L		LH, IU/L		E2, pmol/L	
	Model 1 β (95% CI)	Model 2 β (95% CI)	Model 1 β (95% CI)	Model 2 β (95% CI)	Model 1 β (95% CI)	Model 2 β (95% CI)	Model 1 β (95% CI)	Model 2 β (95% CI)
Pooled (*n* = 3283)								
Q1 (low)	Ref	Ref	Ref	Ref	Ref	Ref	Ref	Ref
Q2 (mild)	0.24 (−0.69, 1.16)	0.02 (−0.91, 0.95)	0.23 (−0.83, 1.31)	0.17 (−0.91, 1.24)	−0.09 (−0.53, 0.35)	−0.13 (−0.58, 0.31)	3.37 (−3.11, 9.85)	3.54 (−2.96, 10.06)
Q3 (moderate)	−0.68 (−1.61, 0.24)	−0.69 (−1.60, 0.22)	0.68 (−0.45, 1.81)	0.59 (−0.55, 1.73)	0.11 (−0.36, 0.58)	0.05 (−0.42, 0.52)	1.64 (−5.22, 8.51)	1.41 (−5.51, 8.32)
Q4 (high)	−0.91 (−1.77, −0.06) *	−0.89 (−1.73, −0.05) *	0.11 (−0.98, 1.21)	−0.04 (−1.17, 1.09)	0.12 (−0.34, 0.57)	0.01 (−0.45, 0.48)	0.09 (−6.55, 6.74)	−0.56 (−7.38, 6.27)
Normal (*n* = 1756)								
Q1 (low)	Ref	Ref	Ref	Ref	Ref	Ref	Ref	Ref
Q2 (mild)	−0.88 (−2.30, 0.55)	−1.03 (−2.44, 0.39)	−0.25 (−2.29, 1.79)	−0.47 (−2.53, 1.58)	−0.30 (−1.09, 0.49)	−0.42 (−1.21, 0.36)	−2.72 (−11.76, 6.32)	−2.21 (−11.33, 6.92)
Q3 (moderate)	−1.14 (−2.55, 0.27)	−1.08 (−2.47, 0.31)	1.22 (−0.99, 3.44)	1.06 (−1.21, 3.32)	−0.18 (−1.04, 0.67)	−0.35 (−1.22, 0.51)	−2.00 (−11.80, 7.80)	−2.66 (−12.70, 7.39)
Q4 (high)	−1.47 (−2.76, −0.19) *	−1.48 (−2.72, −0.24) *	−0.25 (−2.42, 1.92)	−0.42 (−2.65, 1.80)	0.05 (−0.78, 0.89)	−0.13 (−0.98, 0.72)	−4.60 (−14.21, 5.00)	−5.73 (−15.60, 4.14)
Overweight/obese (*n* = 1527)								
Q1 (low)	Ref	Ref	Ref	Ref	Ref	Ref	Ref	Ref
Q2 (mild)	1.10 (−0.10, 2.31)	0.86 (−0.35, 2.08)	0.64 (−0.34, 1.62)	0.59 (−0.40−1.58)	0.09 (−0.40, 0.57)	0.07 (−0.42, 0.56)	3.50 (−5.61, 12.60)	3.46 (−5.96, 12.87)
Q3 (moderate)	−0.28 (−1.49, 0.93)	−0.26 (−1.45, 0.93)	0.25 (−0.78, 1.28)	0.16 (−0.88, 1.19)	0.29 (−0.22, 0.79)	0.25 (−0.26, 0.76)	3.68 (−5.87, 13.22)	3.76 (−5.86, 13.38)
Q4 (high)	−0.35 (−1.48, 0.79)	−0.23 (−1.34, 0.88)	0.38 (−0.60, 1.37)	0.17 (−0.85, 1.18)	0.17 (−0.31, 0.65)	0.08 (−0.42, 0.57)	8.68 (−0.45, 17.82)	8.57 (−0.64, 17.79)

FSH, follicle-stimulating hormone; LH, luteinizing hormone; E2, estradiol. Model 1: adjusted for age and BMI; Model 2: adjusted for age, BMI, smoker, alcoholic drinker, physical activity, and fasting blood glucose. * *p* < 0.05.

**Table 5:** Multivariate linear regression coefficients for sperm parameters in relation to the dietary pattern quartiles according to nutrition status.

Dietary Pattern Quartiles	SC, 10^6^/mL		TSM, %		PRM, %		NSM, %	
	Model 1 β (95% CI)	Model 2 β (95% CI)	Model 1 β (95% CI)	Model 2 β (95% CI)	Model 1 β (95% CI)	Model 2 β (95% CI)	Model 1 β (95% CI)	Model 2 β (95% CI)
Pooled (*n* = 3283)								
Q1 (low)	Ref	Ref	Ref	Ref	Ref	Ref	Ref	Ref
Q2 (mild)	−0.43 (−3.48, 2.62)	−0.37 (−3.46, 2.72)	0.87 (−0.56, 2.29)	0.89 (−0.53, 2.32)	0.24 (−1.59, 2.06)	0.24 (−1.58, 2.06)	−0.72 (−2.36, 0.91)	−0.69 (−2.32, 0.94)
Q3 (moderate)	−2.24 (−5.3, 0.83)	−2.21 (−5.28, 0.87)	−0.20 (−1.60, 1.20)	−0.20 (−1.60, 1.20)	0.32 (−1.46, 2.11)	0.33 (−1.46, 2.12)	−0.55 (−2.16, 1.05)	−0.48 (−2.07, 1.12)
Q4 (high)	−5.54 (−8.67, −2.42) **	−5.55 (−8.68, −2.41) **	−0.41 (−1.80, 0.98)	−0.63 (−2.03, 0.78)	0.05 (−1.73, 1.83)	−0.17 (−1.97, 1.62)	−2.57 (−4.16, −0.98) **	−2.22 (−3.83, −0.62) *
Normal (*n* = 1756)								
Q1 (low)	Ref	Ref	Ref	Ref	Ref	Ref	Ref	Ref
Q2 (mild)	0.55 (−3.57, 4.66)	0.78 (−3.39, 4.95)	1.00 (−0.88, 2.88)	1.04 (−0.84, 2.93)	0.78 (−1.62, 3.19)	0.85 (−1.55, 3.25)	−0.55 (−2.72, 1.61)	−0.60 (−2.75, 1.55)
Q3 (moderate)	−1.99 (−6.09, 2.11)	−1.97 (−6.08, 2.14)	−0.93 (−2.76, 0.91)	−0.92 (−2.77, 0.92)	−0.02 (−2.37, 2.33)	0.06 (−2.29, 2.41)	−0.57 (−2.69, 1.54)	−0.57 (−2.68, 1.53)
Q4 (high)	−4.52 (−8.72, −0.31) *	−4.61 (−8.82, −0.40) *	0.18 (−1.66, 2.02)	−0.08 (−1.94, 1.79)	−0.19 (−2.55, 2.16)	−0.55 (−2.94, 1.83)	−2.23 (−4.35, −0.11) *	−1.64 (−3.77, 0.49)
Overweight/obese (*n* = 1527)								
Q1 (low)	Ref	Ref	Ref	Ref	Ref	Ref	Ref	Ref
Q2 (mild)	−1.50 (−6.06, 3.06)	−1.81 (−6.43, 2.81)	0.72 (−1.47, 2.91)	0.71 (−1.48, 2.90)	0.32 (−2.41, 3.04)	0.29 (−2.47, 3.04)	−0.98 (−3.48, 1.52)	−0.91 (−3.42, 1.59)
Q3 (moderate)	−2.24 (−6.89, 2.40)	−2.31 (−6.96, 2.35)	0.84 (−1.33, 3.00)	0.81 (−1.36, 2.98)	0.77 (−2.01, 3.54)	0.74 (−2.04, 3.52)	−0.47 (−2.94, 2.00)	−0.36 (−2.83, 2.12)
Q4 (high)	−6.73 (−11.42, −2.03) **	−6.73 (−11.44, −2.02) **	−1.11 (−3.24, 1.01)	−1.30 (−3.45, 0.85)	−0.48 (−3.29, 2.33)	−0.52 (−3.32, 2.29)	−3.02 (−5.44, −0.59) *	−2.90 (−5.35, −0.44) *

SC, sperm concentration; TSM, total sperm motility; PRM, progressive motility; NSM, normal sperm morphology. Model 1: adjusted for age and BMI. Model 2: adjusted for age, BMI, smoker, alcoholic drinker, physical activity, and fasting blood glucose. * *p* < 0.05 and ** *p* < 0.01.

## Data Availability

The data that support the findings of this study are available from Mei Jau (M.J.) Health Management Institute, but restricted for research use only. The data are not publicly available. Data are available from the authors upon reasonable request and with permission of MJ Health Management Institute.

## Supplementary Materials

The following are available online at https://www.mdpi.com/2072-6643/13/1/259/s1, Table S1: characteristics of men according to quartiles of dietary pattern.
