# Supplementary material for: Association of Testosterone-Related Dietary Pattern with Testicular Function among Adult Men: A Cross-Sectional Health Screening Study in Taiwan
Source: Nutrients. 2021 Jan 18;13(1):259. doi: 10.3390/nu13010259 (PMC7830687; doi:10.3390/nu13010259)
Supplement: Supplementary file 1 [file nutrients-13-00259-s001.pdf]

Association of testosterone-related dietary pattern with testicular function among adult men: A cross-sectional health screening study in Taiwan

Adi Lukas Kurniawan [1*], Chien-Yeh Hsu [2], Jane C-J Chao [3, 4, 5*], Rathi Paramastri [3], Hsiu-An Lee [6], Pao-Chin Lai [7,8], Nan-Chen Hsieh [1,2], and Shu-Fang Vivienne Wu [1,9]

S1 Table. Characteristics of men according to quartiles of dietary pattern

	Total (n = 3283)	Quartiles of dietary pattern scores				P^{a}
		Q1 (n = 806)	Q2 (n = 842)	Q3 (n = 800)	Q4 (n = 835)	
Age						<0.001
<30 y	1130 (34.4)	240 (21.2)	273 (24.2)	277 (24.5)	349 (30.1)	
31 – 40 y	1527 (46.5)	368 (24.1)	367 (24.1	408 (26.7)	384 (25.1)	
> 41 y	626 (19.1)	198 (31.6)	202 (32.3)	115 (18.4)	111 (17.7)	
Smoker						<0.001
No	2035 (62.0)	554 (27.1)	569 (28.0)	506 (24.9)	406 (20.0)	
Yes	1248 (38.0)	252 (20.1)	273 (21.9)	294 (23.6)	429 (34.4)	
Alcoholic drinker						0.001
No	2584 (78.7)	637 (24.7)	691 (26.7)	651 (25.2)	605 (23.4)	
Yes	699 (21.3)	169 (24.2)	151 (21.6)	149 (21.3)	230 (32.9)	
Physical activity						<0.001
None/light	1844 (56.2)	407 (22.1)	467 (25.3)	463 (25.1)	507 (27.5)	
Moderate/intense	1439 (43.8)	399 (27.7)	375 (26.1)	337 (23.4)	328 (22.8)	
BMI, kg/m^2	24.1 ± 3.4	24.0 ± 3.1	24.0 ± 3.1	24.0 ± 3.5	24.3 ± 3.7	0.09
Body fat, %	23.9 ± 5.4	23.4 ± 5.1	23.9 ± 5.3	23.8 ± 5.5	24.5 ± 5.7	0.001
Leukocytes, $10^3/\mu L$	6.2 ± 1.5	6.1 ± 1.5	6.2 ± 1.5	6.2 ± 1.5	6.4 ± 1.6	<0.001
Neutrophil/lymphocyte ratio	1.8 ± 0.8	1.8 ± 0.8	1.8 ± 0.7	1.8 ± 0.9	1.8 ± 0.7	0.26
Erythrocytes, $10^6/\mu L$	5.2 ± 0.4	5.2 ± 0.4	5.2 ± 0.4	5.2 ± 0.4	5.2 ± 0.4	0.30
Hemoglobin, g/dL	15.4 ± 1.0	15.3 ± 1.0	15.3 ± 0.9	15.4 ± 1.0	15.4 ± 1.0	0.31
Hematocrit, %	45.7 ± 2.8	45.6 ± 2.8	45.7 ± 2.7	45.7 ± 2.7	45.7 ± 2.8	0.43
Fasting glucose, mmol/L	5.5 ± 0.7	5.5 ± 0.5	5.5 ± 0.6	5.5 ± 0.8	5.5 ± 0.8	0.53
Triglycerides, mmol/L	1.4 ± 0.8	1.3 ± 0.8	1.3 ± 0.8	1.3 ± 0.8	1.4 ± 0.9	<0.001
Total cholesterol, mmol/L	5.0 ± 0.9	4.9 ± 0.9	5.0 ± 0.9	5.0 ± 0.9	5.0 ± 0.9	0.08
HDL-cholesterol, mmol/L	1.3 ± 0.3	1.4 ± 0.3	1.3 ± 0.3	1.3 ± 0.3	1.3 ± 0.3	<0.001
LDL-cholesterol, mmol/L	3.1 ± 0.8	3.0 ± 0.8	3.1 ± 0.8	3.1 ± 0.8	3.1 ± 0.8	0.09
TC/HDL-cholesterol ratio	3.9 ± 1.0	3.8 ± 1.0	3.9 ± 1.0	3.8 ± 0.9	4.0 ± 1.0	<0.001
Creatinine, μmol/L	95.3 ± 10.8	95.5 ± 10.6	95.7 ± 10.8	96.0 ± 11.2	94.2 ± 10.6	0.03
eGFR, mL/min/1.73 m^2	90.2 ± 13.0	89.1 ± 12.8	89.2 ± 13.2	90.1 ± 13.0	92.3 ± 13.0	<0.001
C-reactive protein, nmol/L	19.7 ± 32.8	19.7 ± 41.0	21.1 ± 35.7	19.9 ± 28.5	18.1 ± 23.2	0.22
Iron, μmol/L	18.7 ± 6.3	18.8 ± 6.5	18.3 ± 6.1	18.8 ± 6.1	19.0 ± 6.5	0.21
TIBC, μmol/L	55.7 ± 8.0	55.4 ± 8.2	54.9 ± 7.9	55.7 ± 7.4	56.4 ± 8.3	0.27
Transferrin saturation, %	45.0 ± 21.7	43.5 ± 23.6	44.3 ± 21.3	45.2 ± 20.3	46.7 ± 21.7	0.27
Ferritin, μg/L	240.7 ± 151.6	227.3 ± 146.2	212.1 ± 156.3	252.6 ± 143.6	270.4 ± 153.7	0.004
Uric acid, mmol/L	0.4 ± 0.1	0.3 ± 0.1	0.3 ± 0.1	0.4 ± 0.1	0.4 ± 0.1	<0.001
FSH, IU/L	4.6 ± 5.4	4.7 ± 3.1	4.7 ± 3.8	4.8 ± 9.2	4.2 ± 4.0	0.45
LH, IU/L	3.3 ± 2.2	3.3 ± 1.6	3.2 ± 1.5	3.4 ± 3.0	3.4 ± 2.5	0.62
Testosterone, nmol/L	17.5 ± 6.0	17.9 ± 5.9	18.0 ± 6.4	17.3 ± 5.9	16.9 ± 5.8	0.04
E2, pmol/L	90.3 ± 32.8	87.6 ± 27.3	90.5 ± 32.3	91.4 ± 33.8	91.6 ± 38.3	0.03

SC, 10^6/mL	46.6 ± 25.4	48.6 ± 26.5	48.2 ± 25.8	46.4 ± 25.8	43.1 ± 23.1	0.002
TSM, %	67.1 ± 11.6	67.0 ± 11.9	67.9 ± 11.4	66.9 ± 11.5	66.9 ± 11.6	0.41
PRM, %	48.3 ± 14.8	48.0 ± 14.3	48.3 ± 15.7	48.4 ± 14.5	48.4 ± 14.7	0.97
NSM, %	67.0 ± 13.3	67.8 ± 13.6	67.2 ± 13.9	67.4 ± 13.4	65.6 ± 12.4	0.032

BMI, body mass index; HDL-cholesterol, high-density lipoprotein cholesterol; LDL-cholesterol, low-density lipoprotein cholesterol; TC, total cholesterol; eGFR, estimated glomerular filtration rate; TIBC, total iron-binding capacity; FSH, follicle-stimulating hormone; LH, luteinizing hormone; E2, estradiol; SC, sperm concentration; TSM, total sperm motility; PRM, progressive motility; NSM, normal sperm morphology.

Data are expressed in number (%) and mean ± standard deviation (SD) for categorical and continuous variables respectively.

[a] P-value was analyzed using a chi-square test for categorical variables and a general linear model for continuous variables.
